# CircRNAs as biomarkers for osteoporosis: a systematic review and meta-analysis

**DOI:** 10.3389/fendo.2026.1768245

**Published:** 2026-08-17

**Authors:** Xiaoyu Li, Jili Wang, Xiaoge Wang, Shaochong Guo, Shiyu Liu, Ruyin Liu, Haobo Liang, Shangzeng Wang

**Affiliations:** 1 The Second Affiliated Hospital of Henan University of Chinese Medicine, Zhengzhou, China; 2 Henan Provincial Hospital of Traditional Chinese Medicine, Zhengzhou, China; 3 Henan University of Chinese Medicine, Zhengzhou, China

**Keywords:** biomarker, circRNA, diagnostic test accuracy, meta-analysis, osteoporosis

## Abstract

**Background:**

With population ageing, osteoporosis (OP) is increasing worldwide. Circular RNAs (circRNAs) show aberrant expression in OP and may serve as non-invasive biomarkers, but their overall diagnostic performance and sources of heterogeneity remain unclear. This study aimed to systematically evaluate the diagnostic accuracy of circRNAs as potential non-invasive biomarkers for OP and to explore sources of heterogeneity.

**Methods:**

PubMed, Embase, Web of Science, the Cochrane Library, China National Knowledge Infrastructure (CNKI), WanFang, and VIP were searched from inception to 25 November 2025. Studies evaluating circRNAs or circRNA panels for osteoporosis (OP) using DXA-derived bone mineral density (BMD) as the reference standard were included. Methodological quality was assessed using QUADAS-2. A bivariate random-effects model was used to pool sensitivity, specificity, positive and negative likelihood ratios, diagnostic odds ratios, and the area under the summary receiver operating characteristic curve. Subgroup analyses, meta-regression, sensitivity analyses, and Deeks’ funnel plot asymmetry test were performed.

**Results:**

12 articles comprising 14 independent studies (801 OP patients and 640 non-OP controls) were included. The pooled SEN was 0.87 and SPE was 0.79; PLR and NLR were 4.24 and 0.16, respectively, with a DOR of 26.24 and an AUC of 0.91, indicating good overall discriminatory ability. Spearman correlation (r = −0.169, *P* = 0.563) suggested no obvious threshold effect. Subgroup analyses and meta-regression showed that diagnostic performance tended to be better in studies enrolling postmenopausal OP patients, with mean age < 65 years, larger sample sizes, non-serum/plasma samples (peripheral blood mononuclear cells or exosomes), and down-regulated circRNAs. Deeks’ funnel plot indicated no significant publication bias, and sensitivity analyses suggested that the main findings were robust.

**Conclusion:**

CircRNAs demonstrate favorable diagnostic accuracy for osteoporosis and may serve as promising non-invasive biomarkers for screening and risk stratification. Current evidence supports their role as adjunctive tools that complement, rather than replace, DXA-derived bone mineral density assessment and traditional clinical risk factor evaluation. However, they should not be considered stand-alone diagnostic tests. Further large-scale prospective studies are required before routine clinical application can be recommended.

**Systematic Review Registration:**

https://www.crd.york.ac.uk/PROSPERO/, identifier CRD420251240922.

## 1 Introduction

Osteoporosis (OP) is a chronic, systemic skeletal disease characterized by low bone mass and deterioration of bone microarchitecture, which together increase bone fragility and the risk of low-energy fractures (1). As the global population ages, the prevalence of OP is estimated at about 18.3%, rising to 23.1% in women and 11.7% in men, making it a major public health concern in older adults (2). Dual-energy X-ray absorptiometry (DXA), quantitative ultrasound (QUS), and quantitative computed tomography (QCT) are three commonly used measurement methods for diagnosing or assisting in its diagnosis. DXA remains the clinical “gold standard” for diagnosing OP and is widely used to guide fracture risk assessment and treatment decisions. However, bone mineral density (BMD) primarily reflects bone mineral content and is relatively insensitive to changes in trabecular microarchitecture, cortical bone quality and bone remodelling. Some individuals whose BMD has not reached the diagnostic threshold for OP still have a high risk of low-trauma fractures (3, 4). QUS is currently limited to peripheral skeletal sites (e.g., the calcaneus, proximal phalanges, tibial shaft, and radius), and its diagnostic performance—particularly accuracy and specificity—remains inferior to that of DXA. In addition, QUS measurements are susceptible to device-related variability and confounding factors such as soft-tissue thickness. QCT enables separate assessment of trabecular and cortical compartments and provides three-dimensional imaging for quantitative evaluation of trabecular morphology, distribution, and related structural parameters (5, 6), while allowing estimation of bone mineral content. However, QCT is associated with substantially higher radiation exposure than DXA and entails greater equipment requirements and cost, which limit its broader clinical adoption (7). According to International Osteoporosis Foundation (IOF) recommendations, commonly used clinical biomarkers include bone turnover markers (BTMs), such as procollagen type I N-terminal propeptide (PINP) and β-isomerized C-terminal telopeptide of type I collagen (β-CTX-I), as well as regulators of calcium–phosphate metabolism, including parathyroid hormone (PTH), 25-hydroxyvitamin D, and serum calcium and phosphate. Although these biomarkers facilitate early appraisal of bone metabolic activity, aid differential diagnosis, and support treatment monitoring, they do not provide definitive diagnostic evidence when used in isolation. Their interpretation should be integrated with DXA-derived bone mineral density and the overall clinical context, and results may be influenced by multiple pre-analytical and biological factors (e.g., dietary intake and timing of blood sampling) (8, 9). Recent multi-omics and translational research has shown that the development of OP involves not only disturbed bone remodeling but also contributions from oxidative stress, chronic inflammation, cellular senescence, and immune dysregulation (10), underscoring the need for new, easily measurable molecular biomarkers to support earlier detection and more refined risk stratification.

Circular RNAs (circRNAs) are covalently closed, circular non-coding RNAs generated through back-splicing of precursor mRNAs. Lacking both a 5′ cap and 3′ poly(A) tail, circRNAs are relatively resistant to exonuclease degradation and therefore show high stability in tissues and body fluids, with appreciable tissue- and cell-type specificity (11–13). Functionally, circRNAs can act as competitive endogenous RNAs that “sponge” microRNAs (miRNAs) or interact with RNA-binding proteins, thereby modulating osteogenic differentiation of bone marrow mesenchymal stem cells, osteoclastogenesis, and key signalling pathways involved in bone remodeling (e.g., BMP, Wnt/β-catenin, RANKL/OPG). Through these mechanisms, circRNAs can ultimately influence both bone mass and bone quality (14–16). In a range of bone-related diseases and OP models, an expanding set of circRNAs has been shown to be differentially expressed and significantly associated with BMD, bone turnover markers, and fracture risk (11, 12). Clinically, several studies have reported disease-related circRNA expression profiles in peripheral serum or plasma, peripheral blood mononuclear cells (PBMCs), and serum exosomes from patients with OP, with individual or combined circRNAs yielding relatively high values of the area under the summary receiver operating characteristic (SROC) curve (AUC) for discriminating OP from non-OP individuals. Together, these findings suggest that circRNAs may serve as promising non-invasive biomarkers for osteoporosis.

However, most studies evaluating circRNAs for the diagnosis of OP have been single-center, small case-control studies, with substantial variability in the circRNAs examined, the types of samples used, and the cut-off values applied. A diagnostic test accuracy meta-analysis conducted according to contemporary methodological standards is still lacking. Accordingly, following the PRISMA-DTA guideline (17), we systematically searched the literature for clinical studies that used DXA-derived BMD as the reference standard and assessed the diagnostic performance of circRNAs for OP. Using a bivariate random-effects model, we pooled sensitivity, specificity, likelihood ratios, the diagnostic odds ratio, and the AUC, and performed subgroup analyses and meta-regression to investigate potential sources of heterogeneity. Our goal was to provide an evidence-based, comprehensive assessment of the overall diagnostic performance of circRNAs in OP and to clarify their potential role as non-invasive biomarkers in clinical practice.

## 2 Materials and methods

### 2.1 Protocol and registration

This study is a systematic review and diagnostic test accuracy (DTA) meta-analysis. Its design and reporting adhered to the PRISMA-DTA guideline (17, 18). The protocol was prospectively registered in the PROSPERO database (registration number: CRD420251240922).

### 2.2 Literature search strategy

Two investigators independently performed a systematic search of databases, including PubMed, Web of Science, Embase, the Cochrane Library, CNKI, WanFang and VIP. The search period extended from database inception to 25 November 2025. A combination of subject headings and free-text terms was used, and the search strategies were adapted for each database. search terms mainly included “osteoporosis”, “postmenopausal osteoporosis”, “bone loss”, “circular RNA”, “circRNA”, “diagnosis”, “sensitivity”, “specificity”, “ROC”, “AUC” and “diagnostic accuracy”; The detailed search strategies for each database are provided in the **Supplementary Table 1**. We also manually screened the reference lists of included articles to identify additional relevant studies.

### 2.3 Eligibility criteria

Studies were included if they met all of the following criteria: (1) diagnostic accuracy studies (cross-sectional, case–control or cohort) evaluating the diagnostic value of circRNAs or circRNA panels for OP; (2) patients with OP and non-osteoporotic controls (healthy individuals or subjects with normal bone mass/osteopenia), regardless of sex or age; (3) DXA-measured BMD, with OP defined according to WHO criteria or relevant guidelines (e.g. T-score ≤ −2.5, or clearly described as clinical or postmenopausal OP); (4) circRNA with diagnostic performance data, and sufficient information to construct or derive a 2 × 2 contingency table (true positive, false positive, true negative, false negative).

And studies were excluded if any of the following applied: (1) Non-diagnostic studies, such as purely basic experimental research (cell or animal), mechanistic studies, or interventional clinical trials; (2) Studies not using circRNAs as diagnostic markers, or in which circRNAs were only evaluated for prognosis, treatment response or risk factors; (3) Conference abstracts, reviews, meta-analyses, case reports, comments or editorials.

### 2.4 Study selection

All retrieved records were imported into EndNote for management. Two investigators independently screened titles and abstracts to exclude obviously irrelevant studies, followed by full-text review according to the eligibility criteria. Disagreements were resolved by discussion or consultation with a third investigator. The study selection process is summarised in a PRISMA flow diagram.

### 2.5 Data extraction

Using a standardised data extraction form, two investigators independently extracted the following information from each included study: first author, year of publication and country/region; study design; participant characteristics (sample size, mean or median age, sex distribution, type of OP, inclusion and exclusion criteria); index test details (circRNA name(s) and direction of expression, sample type, detection method); and diagnostic accuracy data, including sensitivity (SEN), specificity (SPE), positive likelihood ratio (PLR), negative likelihood ratio (NLR), area under the curve (AUC), diagnostic odds ratio (DOR) and data allowing construction or back-calculation of the 2 × 2 table [true positive (TP), false positive (FP), true negative (TN) and false negative (FN)]. Where TP, FP, TN and FN were not directly reported, they were derived from SEN, SPE and sample size when possible. Information on circRNA detection protocols (e.g., RNA extraction, reverse transcription procedures and data normalisation strategies) and diagnostic thresholds (cut-off values) was also extracted where available. However, due to heterogeneity in reporting across studies, particularly the lack of explicit cut-off values in most studies and the absence of standardised detection procedures, these variables were not included as covariates in the meta-regression analysis. Some original studies evaluated multiple circRNAs or performed subgroup analyses within the same cohort. In such cases, each circRNA or independent subgroup was treated as a separate data point in the meta-analysis. Because individual participant data were unavailable and the number of eligible studies was limited, multilevel meta-analysis or other approaches explicitly accounting for within-study correlations were not performed. To evaluate the potential impact of this assumption, leave-one-out sensitivity analyses and influence diagnostics were subsequently conducted to assess the robustness of the pooled estimates. In subgroup analyses, postmenopausal OP was defined as studies explicitly restricting the population to postmenopausal women with OP. Mixed population referred to studies including men, premenopausal women, or those that did not clearly specify the OP subtype.

### 2.6 Quality assessment

The methodological quality and risk of bias of the included studies were assessed independently by two investigators using the Quality Assessment of Diagnostic Accuracy Studies-2 (QUADAS-2) tool (31). QUADAS-2 consists of four domains: patient selection, index test, reference standard, and flow and timing. Each domain is evaluated in terms of risk of bias and concerns about applicability, and rated as “low”, “high” or “unclear” risk. Disagreements were resolved through discussion or consultation with a third investigator. The results of the quality assessment are presented in graphical form (risk-of-bias graph and summary).

### 2.7 Statistical analysis

Stata 15.1 (StataCorp, College Station, TX, USA) and R4.5.1 (R Foundation for Statistical Computing, Vienna, Austria) were used for statistical analyses. A bivariate random-effects model was applied to jointly pool SEN and SPE and to estimate PLR, NLR, DOR and the AUC of the summary receiver operating characteristic (SROC) curve (32). Spearman correlation between the logit of SEN and the logit of (1−SPE) was used to assess the presence of a threshold effect. If no obvious threshold effect was detected, Cochran’s Q test and the *I²* statistic were used to evaluate non-threshold heterogeneity, with *I²* values of approximately 25%, 50% and 75% considered low, moderate and high heterogeneity, respectively (33, 34). Subgroup analyses and meta-regression were performed using the following prespecified covariates: type of OP (postmenopausal or mixed), mean age (< 65 or ≥ 65 years), sample size (≥ 100 or < 100 cases), sample source (serum/plasma or PBMCs/exosomes) and circRNA expression pattern (up- or down-regulated). Study design and methodological quality were also considered as potential sources of heterogeneity. However, because nearly all included studies adopted a case–control design and exhibited highly similar QUADAS-2 assessment profiles, these variables lacked sufficient between-study variability for reliable quantitative exploration. Therefore, they were not included as covariates in the formal meta-regression analysis. Sensitivity analyses were conducted using a leave-one-out approach to assess the influence of individual studies on the pooled estimates (35, 36). If exclusion of a given study changed the pooled SEN, SPE, AUC or DOR by more than 5%, that study was considered to have a potentially important influence. Deeks’ funnel plot asymmetry test was used to assess small-study effects and potential publication bias. Unless otherwise specified, all tests were two-sided, and *P* < 0.05 was considered statistically significant.

## 3 Results

### 3.1 Study selection

**Figure 1:**
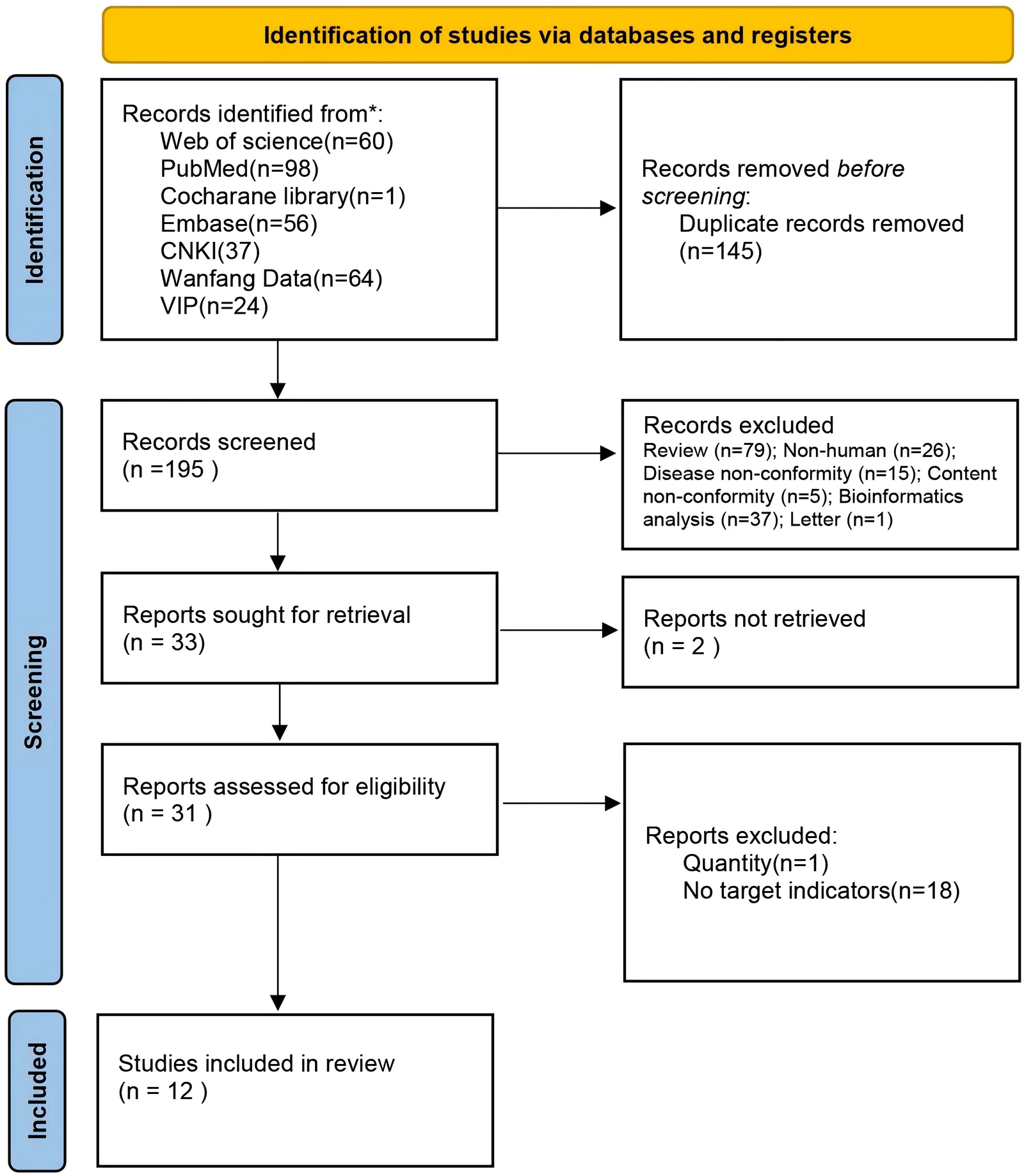
PRISMA flow diagram illustrating the literature search, screening, and study selection process.

The literature search yielded a total of 340 records. After removing duplicates, screening titles and abstracts, and assessing the full texts, 12 articles comprising 14 independent diagnostic studies met the inclusion criteria. The study selection process is summarised in the PRISMA flow diagram (**Figure 1**).

### 3.2 Characteristics of included studies

The main characteristics of the included studies are summarised in **Table 1**. Overall, 1441 participants were included, consisting of 801 OP patients and 640 non-OP patients. Most studies were conducted in China, with one study from Egypt. Study designs were predominantly case–control, and the study populations largely comprised postmenopausal women, although some studies also included men or did not explicitly report menopausal status. All studies used qRT-PCR to detect circRNA expression. Sample types included serum/plasma samples and non-serum/plasma samples, the latter mainly comprising peripheral blood mononuclear cells (PBMCs) and serum-derived exosomes. Target circRNAs included both up-regulated and down-regulated molecules, and several studies evaluated more than one circRNA or reported multiple subgroups.

**Table 1:** Basic characteristics of studies.

Authors	Country	Cases(Male/Female)		Age		Sample used	Validation Method	CircRNA Profile	Sensitivity	Specificity
		OP	Non-OP	OP	Non-OP					
Zhang, 2023(a) (19)	China	50(22/28)	50(26/24)	76 ± 3.7	74.8 ± 5.1	Serum	qRT-PCR	circRNA_0001445 (down)	0.84	0.64
Zhang, 2023 (20)	China	145(60/85)	49(24/25)	77.8 ± 9.85	72.84 ± 8.92	Serum	qRT-PCR	circRNA_0001795(down)	0.806	0.878
		circRNA_0048211(down)	0.841				0.756			
Zhang, 2023(b) (21)	China	81	40	63.82 ± 7.18	57.50 ± 5.48	Serum	qRT-PCR	CircRNA_0048211 (down)	0.816	0.875
		(0/81)	(0/40)							
Fu, 2025 (22)	China	115 (0/115)	108(0/108)	57.82 ± 9.84	56.23 ± 13.23	Serum	qRT-PCR	circ_0004276 (up)	0.822	0.753
Xiang, 2020 (23)	China	84	90	63.51 ± 4.25	61.32 ± 5.62	plasma	qRT-PCR	circ_0001445 (down)	0.976	0.822
		(0/84)	(0/90)							
Yang, 2023 (24)	China	50	50	63.1 ± 4.6	62.4 ± 4.5	plasma	qRT-PCR	circ_0001445 (down)	0.94	0.88
Han, 2020 (25)	China	57	57	62 ± 6	61 ± 7	Serum/plasma	qRT-PCR	circ_0076690 (down)	0.79	0.85
Huang, 2020 (26)	China	40	40	65 ± 9.70	63.2 ± 9.5	Serum/plasma	qRT-PCR	circ_0002060 (up)	0.78	0.69
Guan, 2021 (27)	China	28	25	59.5 ± 4.37	57.0 ± 5.19	PBMCs	qRT-PCR	circ_0021739 (up)	1	0.429
		(0/28)	(0/25)							
Zhao, 2018 (28)	China	58	41	68.88 ± 3.97	64.32 ± 3.15	PBMCs	qRT-PCR	circ_0001275 (up)	0.778	0.66
		(0/58)	(0/41)							
Ismail, 2024 (29)	Egypt	35	30	68 ± 8.34	66 ± 7.15	PBMCs	qRT-PCR	Circ-0091579 (up)	0.88	0.9
		(0/35)	(0/30)				Circ-HIPK3 (down)	0.9	0.77	
Zhi, 2023 (30)	China	58	60	60.67 ± 6.24	58.32 ± 3.14	Exosome of serum	qRT-PCR	circ_0006859 (up)	0.931	0.933
		(0/58)	(0/60)							

### 3.3 Methodological quality

**Figure 2:**
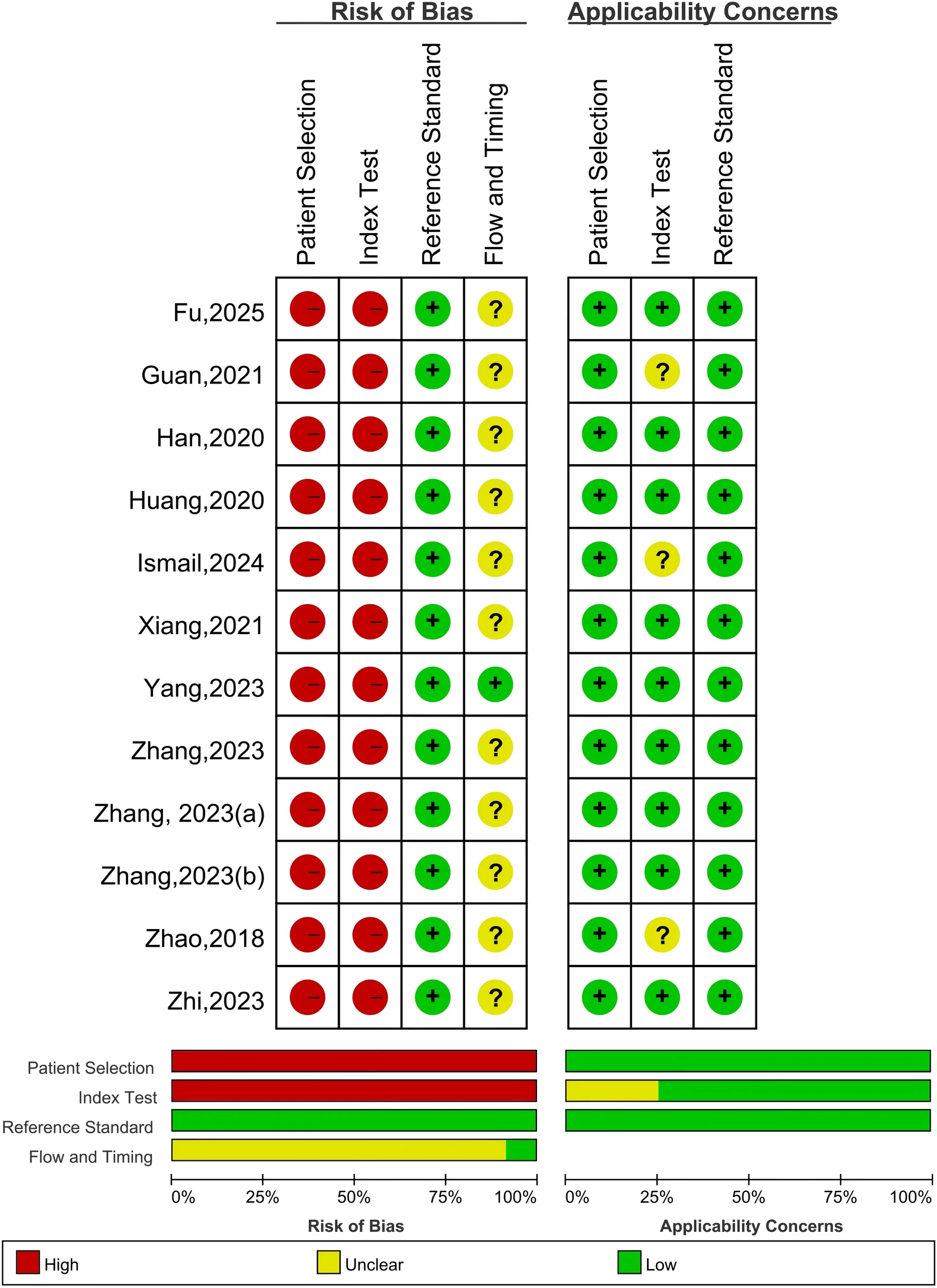
Visual summary of methodological quality based on the QUADAS-2 assessment.

The QUADAS-2 assessment results are shown in **Figure 2**. Most studies were judged to be at low risk of bias in the domains of index test and reference standard. However, in the patient selection and flow and timing domains, several studies had unclear or high risk due to non-consecutive or poorly described enrolment, case–control designs, and lack of clarity regarding the interval between index test and reference standard. In addition, not all studies clearly pre-specified cut-off values, raising the possibility of threshold bias. Overall, the risk of bias was considered acceptable to support pooled analyses, but the results should be interpreted with due caution.

### 3.4 Overall diagnostic accuracy

**Figure 3:**
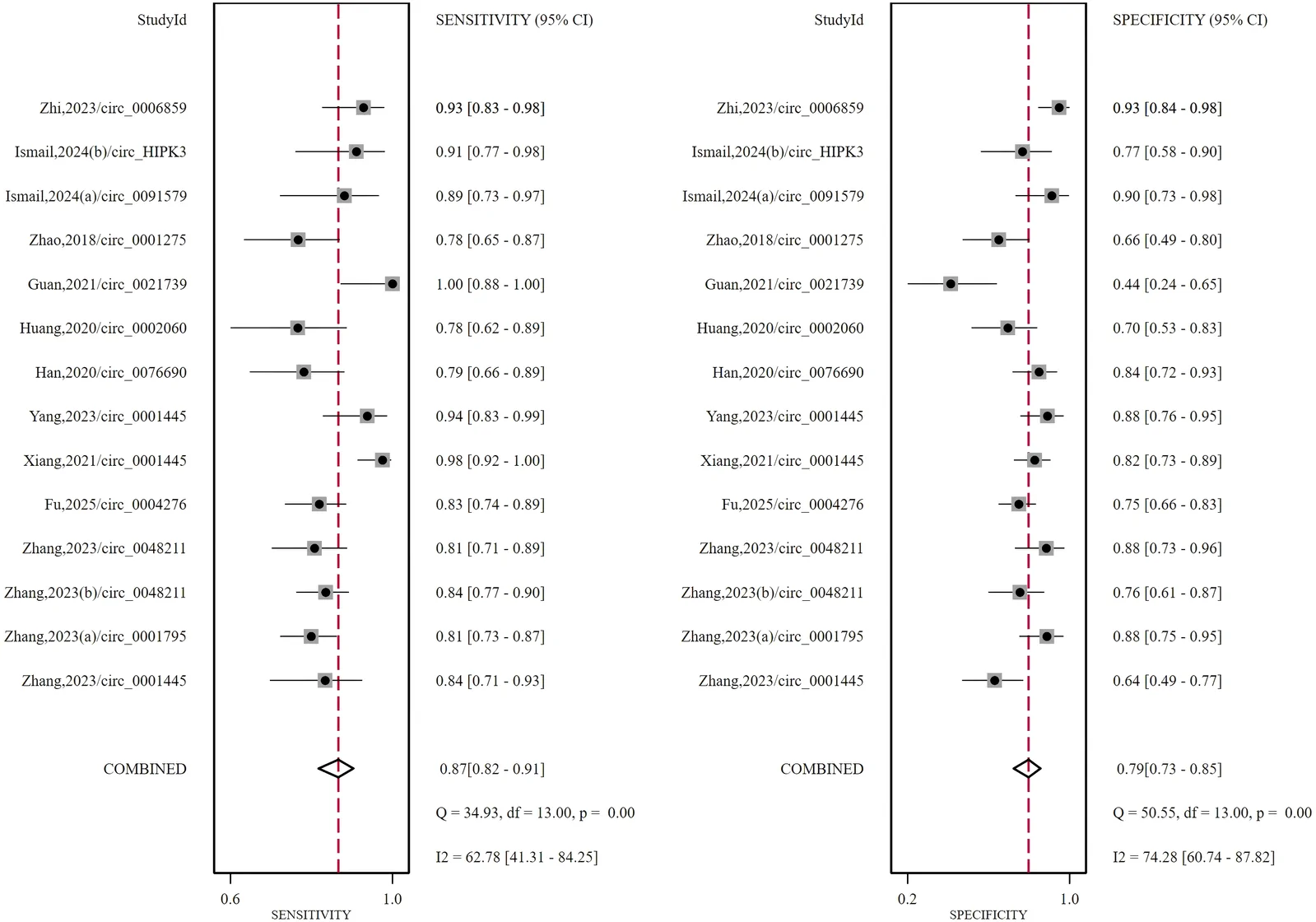
Forest plots of the pooled sensitivity and specificity of circRNAs for the diagnosis of osteoporosis. Point estimates for individual studies and the overall pooled estimates are shown with their 95% confidence intervals.

**Figure 4:**
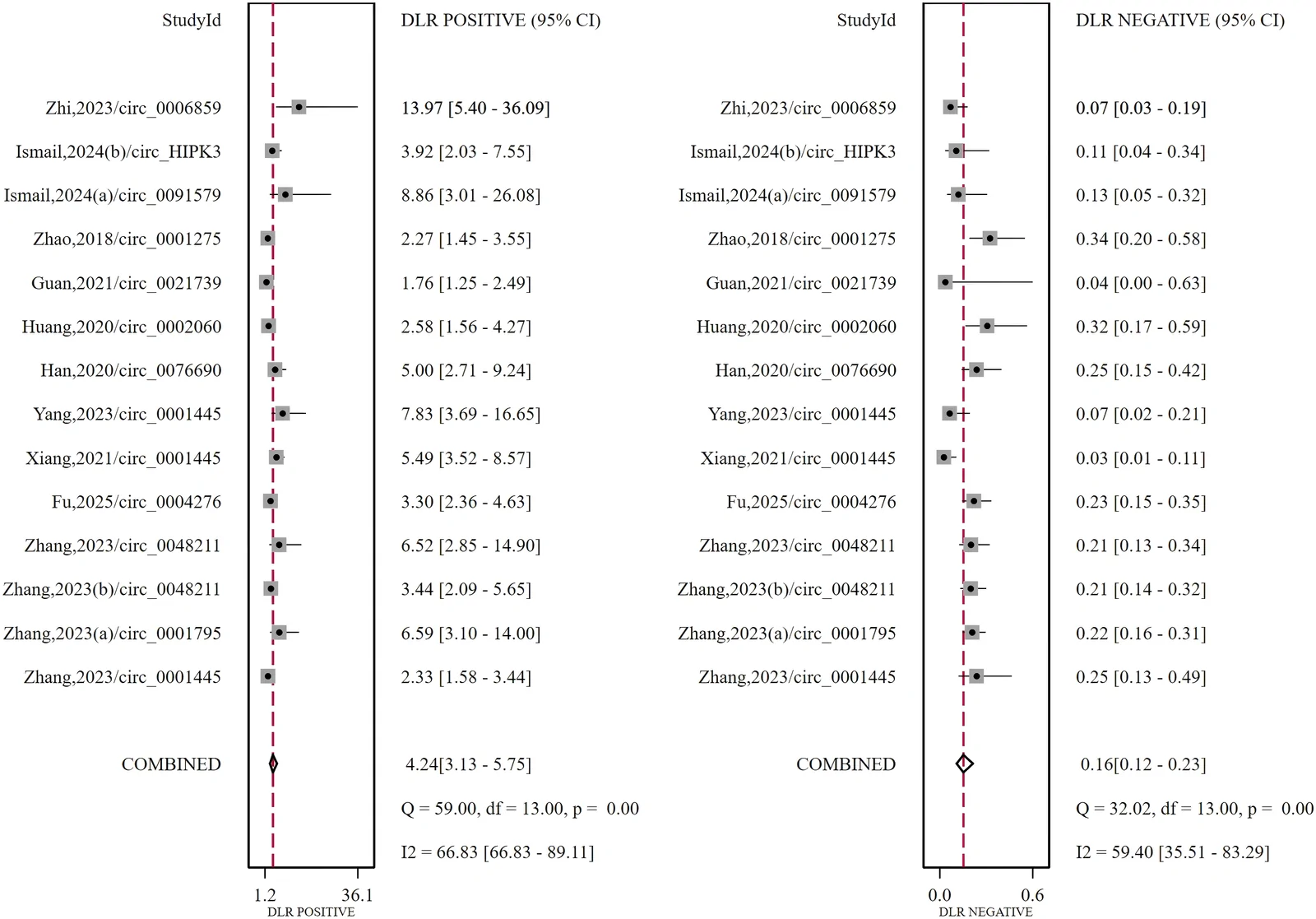
Forest plots of the pooled positive and negative likelihood ratios of circRNAs for the diagnosis of osteoporosis.

**Figure 5:**
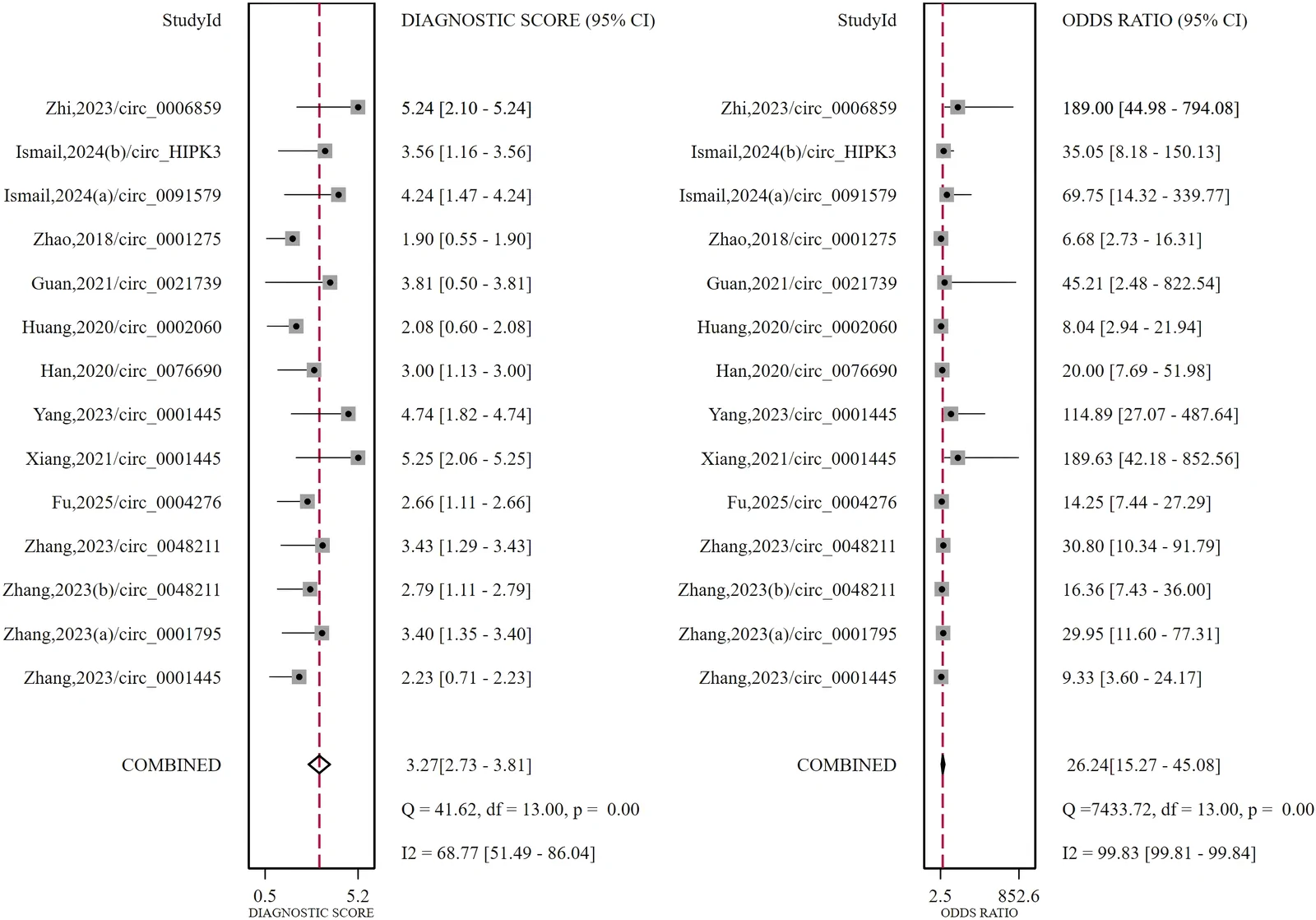
Forest plot of the pooled diagnostic odds ratios for the included studies.

**Figure 6:**
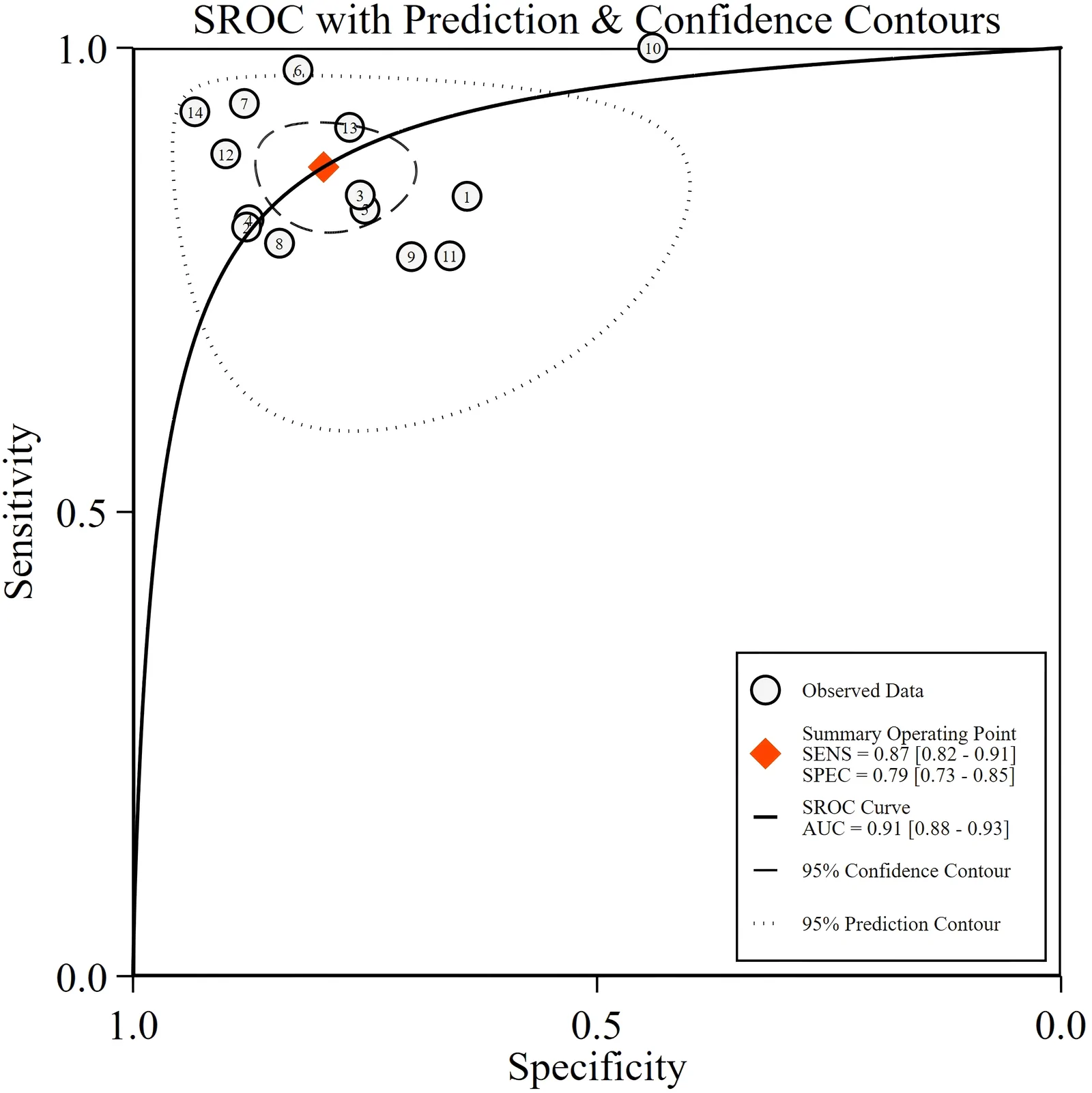
Summary receiver operating characteristic (SROC) curve for the diagnostic accuracy of circRNAs in detecting osteoporosis. Each circle represents one included study, with circle size proportional to its weight. The summary point indicates the pooled sensitivity and specificity; the dashed line shows the 95% confidence region, and the dotted line shows the 95% prediction region.

Under the bivariate random-effects model, circRNAs demonstrated a pooled sensitivity of 0.87 (95% CI: 0.82–0.91) and a pooled specificity of 0.79 (95% CI: 0.73–0.85) for the diagnosis of osteoporosis. The pooled positive likelihood ratio (PLR), negative likelihood ratio (NLR), and diagnostic odds ratio (DOR) were 4.24 (95% CI: 3.13–5.75), 0.16 (95% CI: 0.12–0.23), and 26.24 (95% CI: 15.27–45.08), respectively. The area under the summary receiver operating characteristic (SROC) curve was 0.91, indicating good overall diagnostic performance (**Figures 3**–**6**). Substantial heterogeneity was observed across studies for both sensitivity (*I²* = 79.2%, Cochran’s Q = 57.56, *P* < 0.001) and specificity (*I²* = 75.3%, Cochran’s Q = 52.53, *P* < 0.001), indicating considerable between-study variability.

### 3.5 Threshold effect and heterogeneity

Spearman correlation analysis between the logit of sensitivity and the logit of (1−specificity) yielded r = −0.169 (*P* = 0.563), indicating the absence of a significant threshold effect. However, substantial non-threshold heterogeneity was observed. Cochran’s Q test and *I²* statistics revealed considerable between-study heterogeneity. For sensitivity, Cochran’s Q statistic was 57.56 with an *I²* value of 79.2% (*P* < 0.001). For specificity, Cochran’s Q statistic was 52.53 with an *I²* value of 75.3% (*P* < 0.001). In addition, the overall *I²* was 77%, and the *I²* values for PLR, NLR and DOR ranged from 59.4% to 68.8% (all *P* < 0.01). These findings justified further exploration of heterogeneity using subgroup analyses and meta-regression.

### 3.6 Subgroup and meta-regression analyses

**Figure 7:**
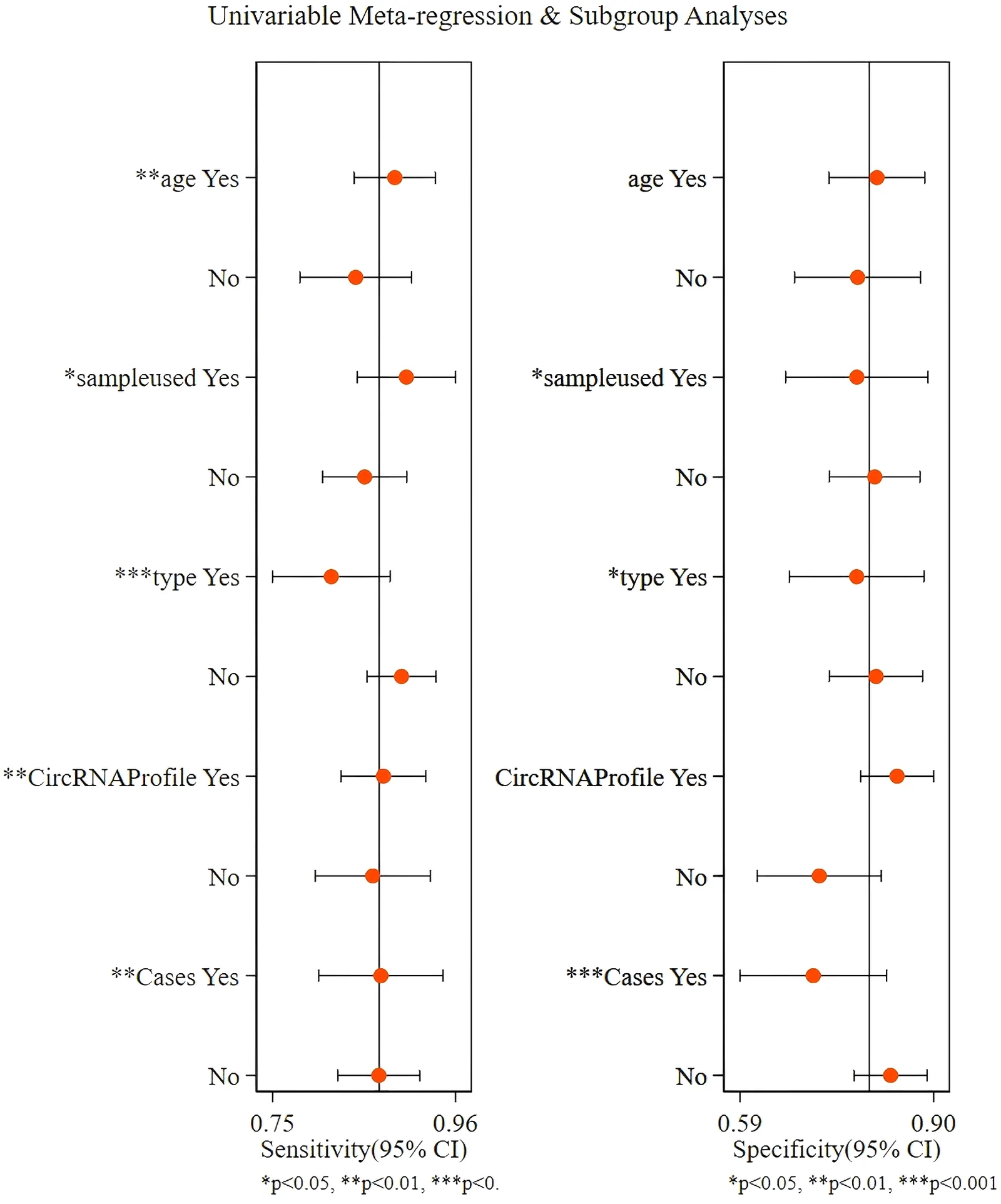
Results of meta-regression and subgroup analyses examining potential sources of heterogeneity in the diagnostic accuracy of circRNAs for osteoporosis. An asterisk next to a covariate indicates a statistically significant difference in diagnostic performance between its subgroups.

Subgroup analyses were conducted according to osteoporosis type (postmenopausal osteoporosis or mixed populations), mean age (≥65 or <65 years), sample size (≥100 or <100 cases), sample source (serum/plasma or non-serum/plasma samples, such as PBMCs or exosomes), and circRNA expression profile (up- or down-regulated). The subgroup results and meta-regression analyses are presented in **Table 2** and **Figure 7**. Overall, relatively higher diagnostic performance was observed in postmenopausal osteoporosis populations, studies with younger mean age, larger sample sizes, non-serum/plasma samples, and down-regulated circRNAs.

**Table 2:** Subgroup analysis.

Subgroup	Cases	Sensitivity 95% CI	Specificity 95% CI	PLR 95% CI	NLR 95% CI	DOR 95% CI	AUC 95% CI
Type of Osteoporosis							
Postmenopausal OP	1018	0.91(0.84-0.95)	0.81(0.71-0.88)	4.72(3.05-7.29)	0.11(0.06-0.20)	41.72(18.8-92.57)	0.93(0.90-0.95)
Mixed OP	682	0.82(0.70-0.85)	0.77(0.68-0.84)	3.59(2.54-5.06)	0.23(0.19-0.29)	15.27(9.41-24.77)	0.84(0.81-0.87)
Mean age							
≥65	717	0.83(0.79-0.86)	0.77(0.68-0.84)	3.63(2.53-5.23)	0.22(0.17-0.28)	16.59(9.76-28.22)	0.84(0.81-0.87)
<65	983	0.90(0.82-0.95)	0.77(0.71-0.88)	4.69(3.03-7.27)	0.12(0.06-0.23)	39.11(16.95-90.22)	0.92(0.90-0.94)
Cases							
≥100	1383	0.87(0.82-0.91)	0.83(0.76-0.87)	4.99(3.59-6.95)	0.16(0.11-0.23)	31.85(16.90-60.00)	0.91(0.89-0.94)
<100	362	0.88(0.76-0.95)	0.70(0.56-0.82)	2.99(1.96-4.57)	0.17(0.08-0.35)	18.10(7.26-45.12)	0.87(0.83-0.89)
Sample							
Serum/Plasma	1300	0.85(0.80-0.89)	0.80(0.74-0.84)	4.20(3.22-5.47)	0.18(0.13-0.26)	22.80(13.30-39.08)	0.89(0.86-0.91)
Non-Serum/Plasma	400	0.91(0.81-0.96)	0.78(0.58-0.90)	4.18(2.02-8.64)	0.11(0.05-0.25)	36.62(11.11-120.67)	0.93(0.91-0.95)
circRNA profile							
UP	638	0.87(0.78-0.92)	0.76(0.61-0.87)	3.69(2.06-6.62)	0.17(0.10-0.30)	21.44(7.89-58.23)	0.90(0.87-0.92)
Down	1062	0.87(0.81-0.92)	0.81(0.76-0.86)	4.68(3.51-6.25)	0.16(0.10-0.24)	30.02(16.61-54.23)	0.90(0.87-0.93)

mixed osteoporosis includes studies enrolling men, premenopausal women or patients without clearly specified osteoporosis subtype. Cases indicates the total number of participants contributed by the studies in each subgroup. Because some studies contributed data to more than one subgroup (e.g., multiple circRNAs or subgroup analyses within the same cohort), the sum of cases across subgroups exceeds the overall sample size.

Meta-regression analyses were subsequently performed separately for sensitivity and specificity to explore potential sources of heterogeneity (**Table 3**). None of the investigated covariates, including age, sample source, osteoporosis type, circRNA expression profile, or sample size, showed a statistically significant association with heterogeneity in either sensitivity or specificity (all *P* > 0.05). Although sample size showed a borderline association with specificity (β = −0.688, 95% CI: −1.479 to 0.102, *P* = 0.082), statistical significance was not reached. These findings suggest that the observed heterogeneity may be multifactorial and not adequately explained by the study-level characteristics examined in the present analysis.

**Table 3:** Meta-regression analysis for sensitivity and specificity.

Covariate	Sensitivity β (95% CI)	*P* value	Specificity β (95% CI)	*P* value
Age	0.209 (-0.486, 0.905)	0.524	0.187 (-0.678, 1.051)	0.646
Sample source	0.279 (-0.501, 1.060)	0.451	-0.210 (-1.119, 0.699)	0.623
Type of OP	-0.449 (-1.082, 0.184)	0.148	-0.159 (-1.044, 0.727)	0.703
circRNA profile	0.094 (-0.623, 0.811)	0.779	0.363 (-0.476, 1.202)	0.364
Sample cases	-0.126 (-0.903, 0.651)	0.730	-0.688 (-1.479, 0.102)	0.082

All covariates were entered as binary variables: Age (<65 vs ≥65 years), Sample source (serum/plasma vs non-serum/plasma), Type of OP (mixed vs postmenopausal), circRNA profile (up-regulated vs down-regulated), and sample size (<100 vs ≥100 cases).

### 3.7 Sensitivity analyses

**Figure 8:**
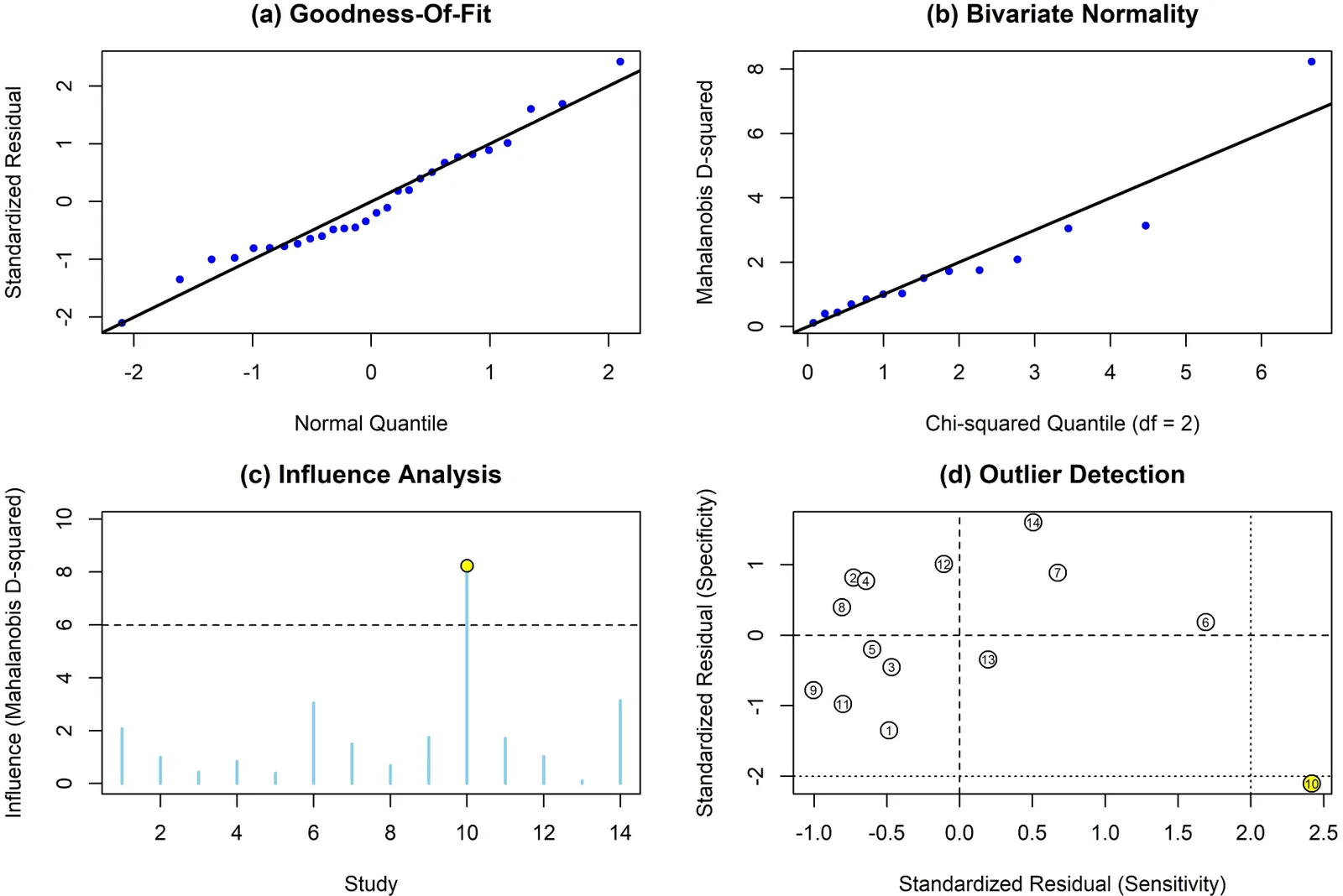
Results of sensitivity analyses. **(A)** Goodness-of-fit plot; **(B)** Bivariate normality plot; **(C)** Influence analysis; **(D)** Outlier detection.

Leave-one-out sensitivity analyses were performed to assess the influence of individual studies on the pooled results (**Figure 8**). Study 10 (27) had the largest influence. Excluding this study yielded a pooled SEN of 0.86 and a pooled SPE of 0.81; the change in SPE exceeded the prespecified 5% threshold, suggesting a notable impact on specificity. However, the AUC decreased only slightly from 0.91 to 0.90 and the DOR from 26.24 to 26.10, both changes being < 5%, indicating that the overall discriminatory ability remained stable (**Table 4**) (37). Similar analyses excluding Xiang, 2020 or both studies simultaneously did not materially alter the main findings. These findings also indirectly support the robustness of the results despite the potential violation of independence assumptions arising from the inclusion of multiple datasets from the same study.

**Table 4:** Pooled diagnostic accuracy and leave-one-out sensitivity analysis.

Index	SEN 95% CI	SPE 95% CI	DOR 95% CI	AUC 95% CI	*I²*
Pooled statistics	0.87(0.82-0.91)	0.79(0.73-0.85)	26.24(15.27-45.08)	0.91(0.88-0.93)	77%
Eliminate Xiang,2020	0.85(0.81-0.88)	0.79(0.72-0.85)	21.28(12.82-35.33)	0.89(0.85-0.91)	75%
Eliminate Guan,2021	0.86(0.82-0.90)	0.81(0.75-0.85)	26.10(15.19-44.84)	0.90(0.88-0.93)	0%
Eliminate Xiang,2020+Guan,2021	0.84(0.81-0.87)	0.81(0.75-0.86)	22.00(13.45-36.78)	0.89(0.86-0.91)	12%

### 3.8 Publication bias and outlier analysis

**Figure 9:**
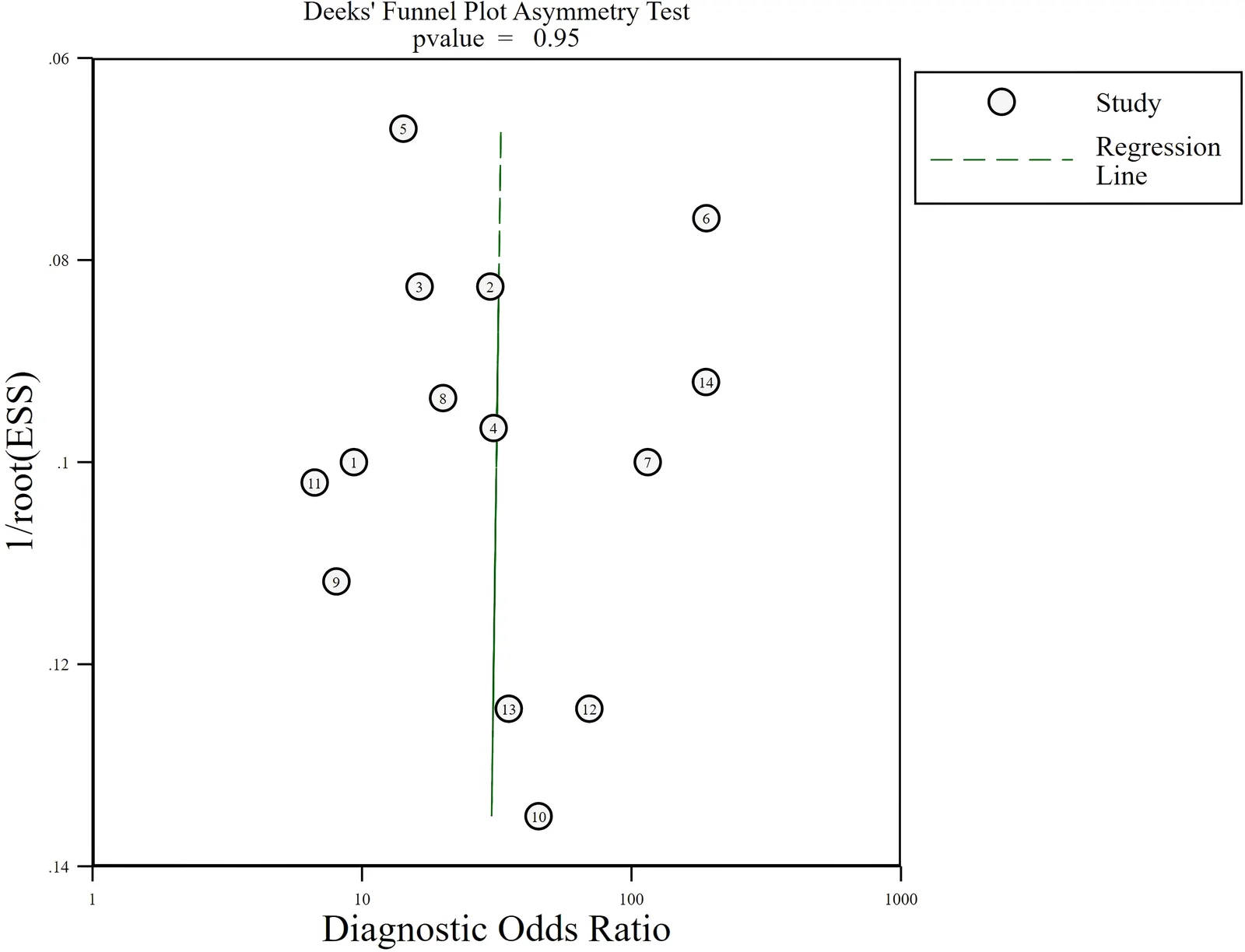
Deeks’ funnel plot for assessing publication bias in studies of circRNAs for the diagnosis of osteoporosis.

**Figure 10:**
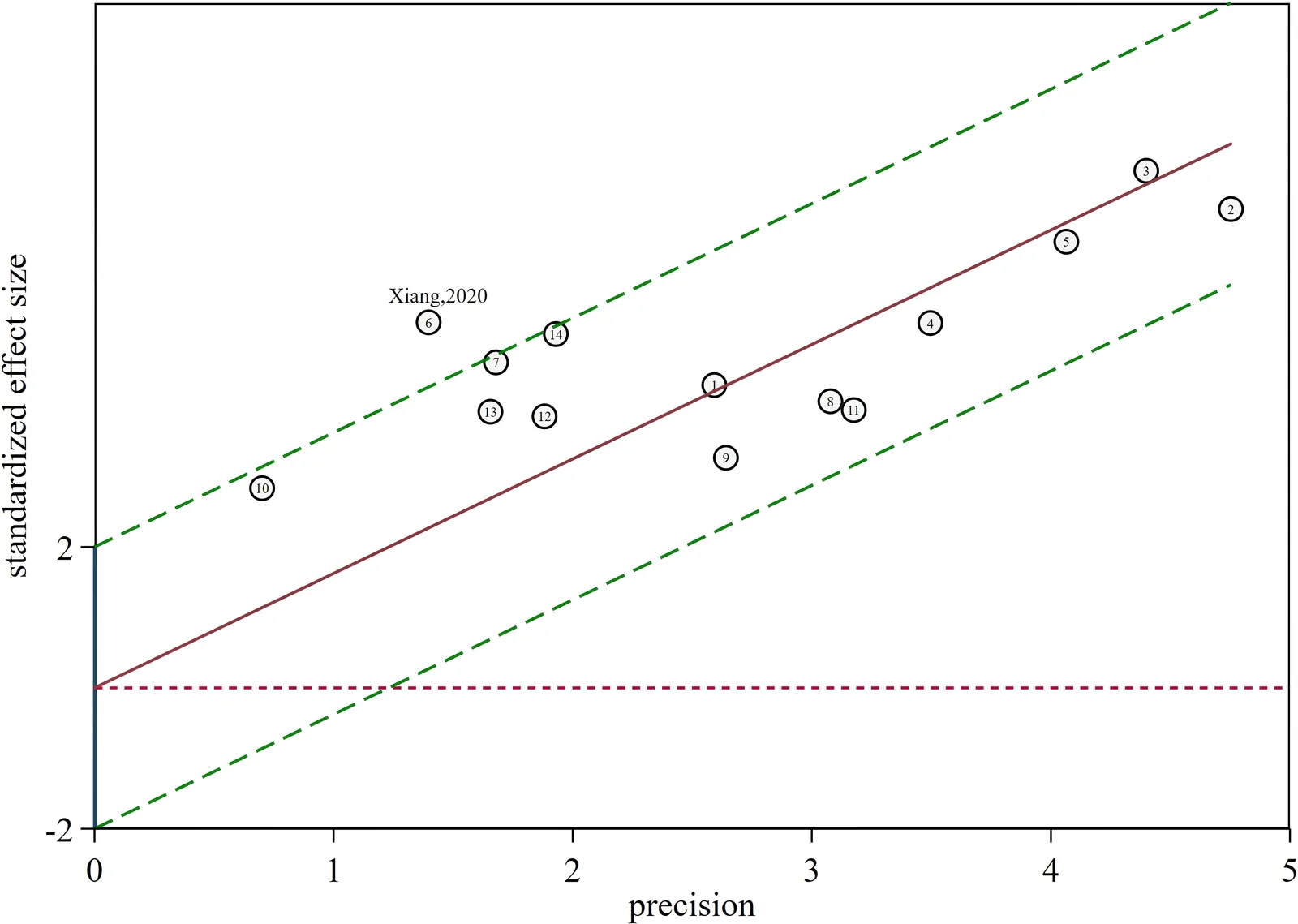
Galbraith plot assessing heterogeneity across studies included in the diagnostic meta-analysis of circRNAs for osteoporosis. The solid diagonal line represents the pooled estimate, and the two dashed lines indicate the 95% confidence interval around the summary effect. Studies lying close to the central line are consistent with the overall estimate, whereas those falling outside the confidence region are potential outliers contributing to heterogeneity.

Deeks’ funnel plot was used to assess publication bias (**Figure 9**). The funnel plot appeared approximately symmetrical, and the regression test yielded *P* > 0.05, suggesting no significant publication bias. Galbraith plots (**Figure 10**) indicated that Xiang, 2020 deviated from the other studies, suggesting it may be an outlier and an important source of heterogeneity. After excluding this study, the pooled SEN was 0.85, SPE 0.79, DOR 21 and AUC 0.89, with *I²* decreasing to 75%. Compared with the primary analysis, these changes were not clinically meaningful, and the diagnostic performance remained generally similar. Therefore, all 14 studies were retained in the final pooled analysis.

### 3.9 Clinical utility

**Figure 11:**
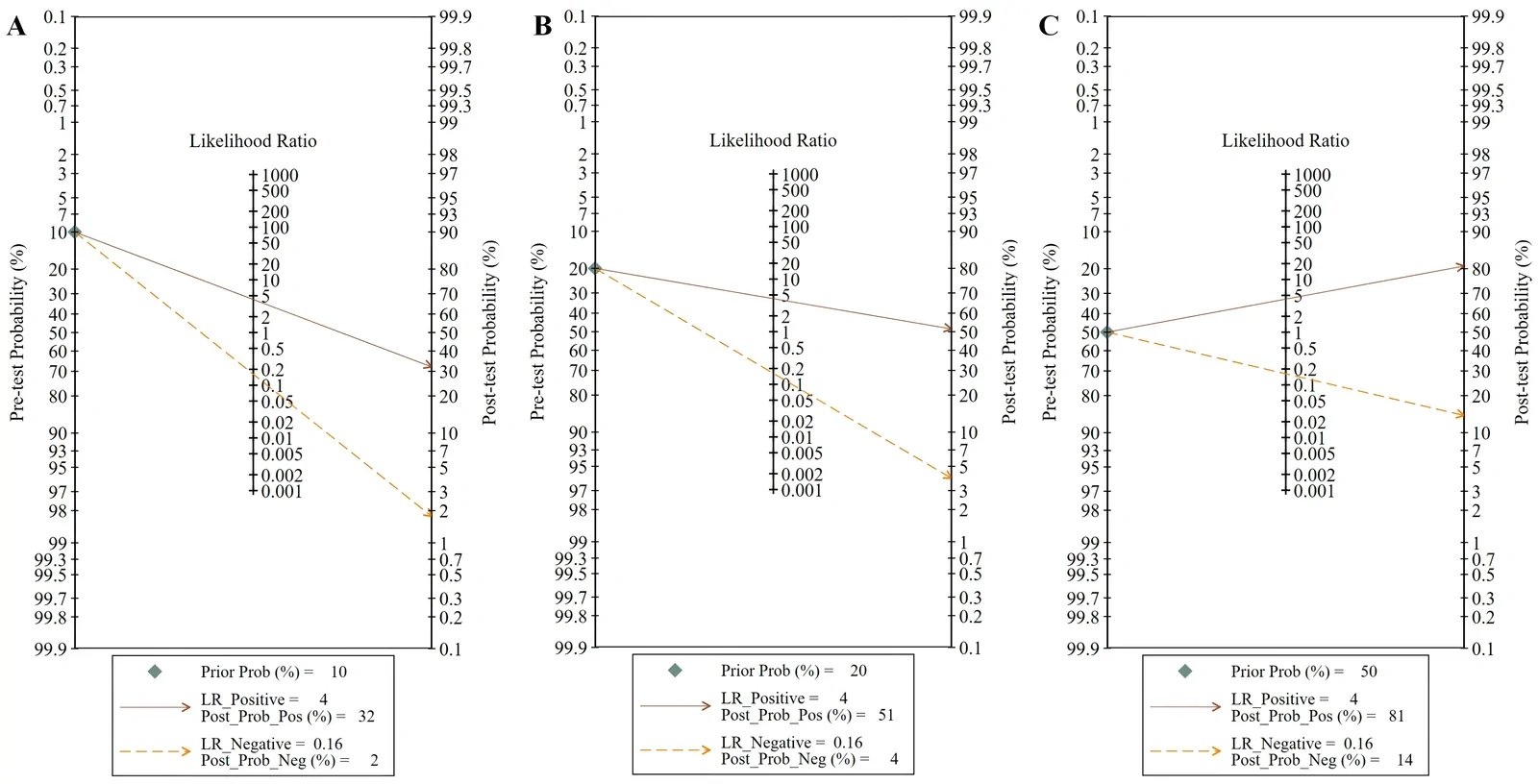
Three Fagan nomogram charts labeled **(A, B)** and **(C)** illustrate changes in pre-test and post-test probabilities based on likelihood ratios for a diagnostic test. Panel **(A)** shows a pre-test probability of 10%, Panel **(B)** shows a pre-test probability of 20%, and Panel **(C)** shows a pre-test probability of 50%.

Fagan’s nomogram was constructed based on the pooled PLR and NLR to assess the clinical utility of circRNAs (**Figure 11**). At a pre-test probability of 10%, a positive circRNA test increased the post-test probability of osteoporosis (OP) to approximately 32%, whereas a negative result reduced it to about 2%. In contrast, at a pre-test probability of 50% (representing a high-risk clinical population), a positive circRNA test increased the post-test probability to approximately 81%, whereas a negative result reduced it to about 14%.

These findings indicate that the clinical utility of circRNAs depends substantially on the baseline risk of OP. In lower-risk settings, although a positive test increases disease probability, the post-test probability remains insufficient to support a definitive diagnosis. Therefore, circRNAs should not be regarded as stand-alone diagnostic tools. In contrast, in higher-risk populations, circRNAs demonstrate greater clinical utility by substantially increasing the likelihood of disease following a positive test result. Accordingly, circRNAs may be more appropriately considered adjunctive biomarkers for screening and risk stratification and should be interpreted in conjunction with DXA-derived BMD and established clinical risk factors.

## 4 Discussion

This meta-analysis synthesised current evidence regarding the performance of circRNAs for diagnosing OP. Based on 12 articles and 14 studies, circRNAs demonstrated good overall diagnostic accuracy, with a pooled SEN of 0.87, SPE of 0.79 and an AUC of 0.91. PLR and NLR values of 4.24 and 0.16, respectively, indicate “moderate” diagnostic utility according to commonly accepted interpretation thresholds (38–40). In a high-risk population with a pre-test probability of 50%, a positive circRNA test increases the post-test probability to ~81%, whereas a negative test reduces it to ~14% (41, 42). The choice of pre-test probability has a substantial impact on post-test estimates. While a value of 50% may reflect high-risk clinical populations, lower pre-test probabilities more representative of general screening settings yield more modest post-test probabilities. These findings highlight the importance of clinical context when interpreting the diagnostic value of circRNAs. The Fagan nomogram further demonstrated that the clinical utility of circRNAs varies substantially according to the pre-test probability of osteoporosis. In a low-risk population with a pre-test probability of 10%, a positive circRNA result increased the post-test probability to only approximately 32%, which is insufficient to support a definitive diagnosis. Therefore, circRNAs should not be regarded as stand-alone diagnostic tools. Instead, they may be more appropriately considered adjunctive biomarkers for screening and risk stratification. Their clinical value appears to be greater in individuals with a higher pre-test probability of osteoporosis, where positive test results substantially increase the likelihood of disease. Thus, circRNAs appear promising as auxiliary tools for screening and risk stratification in high-risk OP populations, but are not yet suitable as stand-alone diagnostic tests.

Heterogeneity is inevitable in diagnostic meta-analyses (33, 34). In our analysis, no significant threshold effect was detected, whereas substantial non-threshold heterogeneity was observed for both sensitivity and specificity. Subgroup analyses suggested that sample source and OP type might contribute to variations in diagnostic performance. Studies using PBMCs or exosomes tended to show higher sensitivity and slightly better overall performance than serum/plasma-based studies, while specificity remained similar (27, 30). However, this finding should be interpreted with caution. PBMC- and exosome-based assays involve more complex sample processing procedures and may be more susceptible to variability in pre-analytical conditions. In addition, differences in patient selection, disease severity, and laboratory protocols may have contributed to the observed subgroup differences. Since sample source was not identified as a significant contributor to heterogeneity in the meta-regression analysis, the apparent superiority of non-serum/plasma samples may reflect methodological or pre-analytical factors rather than a genuine biological advantage. Further well-designed studies using standardized protocols are required to clarify this issue. Similarly, studies in postmenopausal OP populations showed relatively higher specificity, reflecting the more homogeneous clinical characteristics of this population. However, these findings were not supported by meta-regression analysis. Neither sample source nor OP type was identified as a statistically significant contributor to heterogeneity (all P > 0.05). This discrepancy may be attributed to the limited number of included studies and insufficient statistical power. Therefore, these factors should be interpreted as potential rather than confirmed sources of heterogeneity. In addition, the observed heterogeneity is likely to be multifactorial and may not be fully captured by the covariates included in the meta-regression. Methodological differences across studies, including variations in RNA extraction procedures, internal reference selection, and particularly the determination of cut-off values, may also contribute to residual heterogeneity. Although study design and methodological quality are recognised sources of heterogeneity in diagnostic accuracy studies, meaningful subgroup analysis or meta-regression based on these variables was not feasible in the present study because nearly all included studies adopted a case–control design and exhibited highly similar QUADAS-2 assessment profiles. Therefore, these variables lacked sufficient between-study variability for reliable quantitative exploration. Nevertheless, study design and methodological quality may still contribute to residual heterogeneity and should be considered when interpreting the pooled estimates. Therefore, the sources of heterogeneity in this study should be interpreted as complex and multifactorial. Despite the presence of substantial heterogeneity, the pooled estimates derived from the bivariate random-effects model may still provide a summary measure of overall diagnostic performance by accounting for between-study variability. However, these results should be interpreted as an average effect across heterogeneous settings rather than a universally applicable estimate.

We also observed that larger sample sizes and younger mean age (< 65 years) were associated with higher specificity, which may be related to more stable estimates and fewer confounders such as comorbidities and polypharmacy. In addition, down-regulated circRNAs displayed slightly higher specificity than up-regulated circRNAs, suggesting that molecules associated with suppressed osteogenesis or enhanced bone resorption may be more diagnostically informative (11, 13, 43, 44). A possible biological explanation is that some of these circRNAs function as regulators of osteogenic differentiation rather than merely serving as passive biomarkers. For example, hsa_circ_0001445 has been shown to promote osteogenic differentiation and inhibit adipogenic differentiation of hBMSCs by sponging miR-127-5p, thereby regulating the osteogenesis–adipogenesis balance. Similarly, hsa_circ_0076690 enhances osteogenic differentiation through the miR-152/RUNX2 axis. Therefore, the downregulation of such circRNAs may more directly reflect the suppression of osteoblast differentiation, a central event in osteoporosis pathogenesis. This may partly explain why some downregulated circRNAs showed better diagnostic performance (24, 25).

Sensitivity analyses and outlier detection further supported the robustness of our findings. Although studies (23, 27)(e.g. Guan, 2021; Xiang, 2020) had noticeable influence on the pooled estimates, exclusion of these studies did not materially alter the overall diagnostic performance, especially with regard to AUC and DOR, whose changes remained within 5%. This indicates that the main conclusions of this meta-analysis are relatively stable despite the presence of heterogeneity (35, 36).

The present study has several notable strengths. First, we focused on diagnostic test accuracy using DXA-derived BMD as the reference standard, which is critical for evaluating the clinical utility of circRNAs. Second, we used a bivariate random-effects model, which is the recommended approach for pooling sensitivity and specificity in diagnostic meta-analyses (32). Third, we adhered to the PRISMA-DTA guideline and applied the QUADAS-2 tool to systematically assess methodological quality, and we explored heterogeneity through prespecified subgroup analyses and meta-regression.

However, there are several limitations. The number of included studies was limited, and most were case–control or single-centre studies from China, restricting generalisability. Notably, the predominance of case–control designs in the included studies may lead to an overestimation of diagnostic accuracy. In such designs, cases and controls are often selected from clearly defined disease and non-disease populations, which does not fully reflect the spectrum of patients encountered in real clinical settings, thereby introducing spectrum bias. Similarly, the use of single-centre study populations may further limit representativeness and increase the risk of selection bias. These methodological features may partly explain the relatively high sensitivity, specificity and AUC values observed in the present meta-analysis, and thus the results should be interpreted with caution. Another important limitation is the restricted generalisability of the findings. Most of the included studies were conducted in China, and the study populations were predominantly postmenopausal women. Although a small number of studies were conducted in other regions, the overall evidence remains heavily concentrated in a single ethnic and geographical context. Therefore, the applicability of the present results to other ethnic groups, male populations, or secondary osteoporosis remains uncertain. However, postmenopausal osteoporosis represents the most common subtype of osteoporosis and a major target population for early diagnosis and intervention. Thus, focusing on this population may provide clinically relevant insights. In addition, to our knowledge, this study is among the first to systematically evaluate the diagnostic performance of circRNAs in osteoporosis using a quantitative meta-analytic approach. Therefore, despite the limited generalisability, the present findings provide preliminary but important evidence supporting the potential role of circRNAs as diagnostic biomarkers for osteoporosis. Many studies lacked detailed reporting of enrolment procedures, flow and timing, and cut-off selection, raising concerns about selection and threshold bias. Methodological variability across studies represents an important limitation. Although qRT-PCR was the predominant detection method, differences in RNA extraction procedures, reverse transcription protocols, primer design, internal reference selection and data normalisation strategies may affect the comparability of circRNA expression levels. Furthermore, the reporting of diagnostic thresholds (cut-off values) was limited. Only a small number of studies explicitly provided cut-off values, while most did not report the criteria used for threshold determination. In the reported cases, cut-off values were typically derived from study-specific ROC analyses without external validation. This lack of transparency and standardisation may limit reproducibility and contribute to uncertainty in interpreting the pooled estimates. In particular, circRNAs in the included studies were derived from different biological sources, including serum, plasma, peripheral blood mononuclear cells (PBMCs), and serum-derived exosomes, and substantial variability existed in pre-analytical handling, RNA extraction procedures, internal reference selection and data normalisation strategies. These inconsistencies may compromise inter-study comparability and potentially contribute to the observed heterogeneity. Some studies evaluated multiple circRNAs or subgroups within the same cohort, and we treated each as a separate data point. This approach may violate the assumption of statistical independence, as data derived from the same study population are likely to be correlated. Consequently, it may introduce bias, potentially leading to an overestimation of diagnostic performance and an underestimation of the true uncertainty, reflected by artificially narrower confidence intervals. However, this strategy is commonly adopted in diagnostic test accuracy meta-analyses when individual-level data are unavailable, in order to maximise data utilisation and avoid the exclusion of potentially informative markers. Importantly, our sensitivity analyses indicated that the pooled estimates remained stable after sequential exclusion of individual studies, suggesting that the influence of this issue on the overall results is limited. Nevertheless, the findings should be interpreted with caution, and future studies are warranted to validate these results using appropriately designed analyses that account for within-study correlations. Although multilevel meta-analysis may provide a more rigorous framework for handling correlated effect estimates, its application was not feasible in the present study because of the limited number of eligible studies and the lack of individual-level data. In addition, confounding factors such as medication use, metabolic diseases, inflammatory status and malignancy were not consistently controlled for or reported. Finally, most studies used cross-sectional designs, limiting inference about temporal relationships and the prognostic value of circRNAs.

Future research should prioritise large-sample, multicentre, prospective diagnostic accuracy studies in diverse populations, with rigorous study design, appropriate blinding, and pre-specified cut-offs. In particular, the development of standardised workflows for circRNA detection is essential to improve reproducibility and facilitate clinical translation. For circulating samples such as serum and plasma, key pre-analytical variables—including blood collection conditions, centrifugation protocols, platelet depletion procedures and storage conditions—should be carefully harmonised. For PBMC-based analyses, standardised protocols for cell isolation and processing time should be established, whereas for exosomal circRNAs, consistent methodologies for exosome isolation, purity assessment and source validation are required. In addition, consensus on RNA extraction procedures, internal control selection and data normalisation strategies should be established, and diagnostic thresholds should be defined using rigorous and preferably externally validated approaches. Furthermore, representative circRNAs identified in the present meta-analysis could be incorporated into multimarker models that combine miRNAs, lncRNAs, protein biomarkers and clinical risk scores (e.g. FRAX), and evaluated using modern statistical and machine-learning methods, followed by external validation. Mechanistic, functional, and longitudinal studies are also needed to clarify the role of circRNAs in OP pathogenesis and to evaluate their potential in treatment response monitoring and re-fracture risk prediction (30).

## 5 Conclusion

In conclusion, current evidence suggests that circRNAs demonstrate favorable diagnostic accuracy for osteoporosis, with pooled sensitivity and specificity of 0.87 and 0.79, respectively, and an AUC of approximately 0.91. However, the moderate likelihood ratios and Fagan analysis indicate that circRNAs alone are insufficient for establishing or excluding a diagnosis of osteoporosis in routine clinical practice and therefore should not be considered stand-alone diagnostic tools. Instead, circRNAs may serve as promising non-invasive molecular biomarkers for screening and risk stratification, particularly in individuals at increased risk of osteoporosis. Current evidence supports their role as adjunctive tools that complement, rather than replace, DXA-derived bone mineral density assessment and traditional clinical risk factor evaluation. Although diagnostic performance appeared relatively more favorable in postmenopausal populations and in studies using PBMC/exosome samples, these findings should be interpreted with caution. Furthermore, the available evidence is primarily derived from small-sample case–control studies with limited geographic and population diversity. Therefore, large-scale, multicentre, prospective studies are still required to validate these findings before routine clinical implementation can be recommended.

## Data Availability

The original contributions presented in the study are included in the article/**Supplementary Material**. Further inquiries can be directed to the corresponding author.
