# Supplementary material for: CircRNAs as biomarkers for osteoporosis: a systematic review and meta-analysis
Source: Front Endocrinol (Lausanne). 2026 Aug 17;17:1768245. doi: 10.3389/fendo.2026.1768245 (PMC13527778; doi:10.3389/fendo.2026.1768245)
Supplement: Supplementary file 1 [file Table1.docx]

Supplement Table1 Search strategy

| Database | | Search Strategy | |
| --- | --- | --- | --- |
| Pubmed | #1 | | "Osteoporosis"[Mesh] |
|  | #2 | | (((((osteoporosis[Title/Abstract]) OR (osteoporotic[Title/Abstract])) OR (osteopenia[Title/Abstract])) OR ("bone mineral density"[Title/Abstract])) OR ("bone loss"[Title/Abstract])) OR ("low bone mass"[Title/Abstract]) |
|  | #3 | | "RNA, Circular"[Mesh] |
|  | #4 | | ((((circRNA[Title/Abstract]) OR (circRNAs[Title/Abstract])) OR ("circular RNA"[Title/Abstract])) OR ("circular RNAs"[Title/Abstract])) OR ("circularized RNA"[Title/Abstract]) |
|  | #5 | | ((((((((((sensitiv*[Title/Abstract]) OR (specificit*[Title/Abstract])) OR ("sensitivity and specificity"[Title/Abstract])) OR ("predictive value of tests"[Title/Abstract])) OR ("predictive value*"[Title/Abstract])) OR ("likelihood ratio*"[Title/Abstract])) OR ("receiver operating characteristic"[Title/Abstract])) OR (ROC[Title/Abstract])) OR (AUC[Title/Abstract])) OR (diagnos*[Title/Abstract])) OR (accuracy*[Title/Abstract]) |
|  | #6 | | #1 OR #2 |
|  | #7 | | #3 OR #4 |
|  | #8 | | #6 AND #7 |
|  | #9 | | #8 AND #5 |
| CNKI | SU= | | ( 骨质疏松症 + 骨质疏松 + 绝经后骨质疏松 + 骨质减少 + 骨量减少 + 骨密度 ) AND （环状RNA + 环形RNA + 环状核糖核酸 + circRNA） |
